# Predicting Parkinson disease in the community using a nonmotor risk score

**DOI:** 10.1007/s10654-016-0130-1

**Published:** 2016-02-22

**Authors:** Sirwan K. L. Darweesh, Peter J. Koudstaal, Bruno H. Stricker, Albert Hofman, Ewout W. Steyerberg, M. Arfan Ikram

**Affiliations:** 1 Department of Epidemiology, Erasmus MC University Medical Center Rotterdam, Dr. Molewaterplein 50, 3015 GE Rotterdam, The Netherlands; 2 Department of Neurology, Erasmus MC University Medical Center Rotterdam, Rotterdam, The Netherlands; 3 Inspectorate for Health Care, The Hague, The Netherlands; 4 Department of Public Health, Erasmus MC University Medical Center Rotterdam, Rotterdam, The Netherlands; 5 Department of Radiology, Erasmus MC University Medical Center Rotterdam, Rotterdam, The Netherlands

**Keywords:** Parkinson disease, Population-based, Risk prediction, Risk factors

## Abstract

At present, there are no validated methods to identify persons who are at increased risk for Parkinson Disease (PD) from the general population. We investigated the clinical usefulness of a recently proposed non-motor risk score for PD (the PREDICT-PD risk score) in the population-based Rotterdam Study. At baseline (1990), we constructed a weighted risk score based on 10 early nonmotor features and risk factors in 6492 persons free of parkinsonism and dementia. We followed these persons for up to 20 years (median 16.1 years) for the onset of PD until 2011. We studied the association between the PREDICT-PD risk score and incident PD using competing risk regression models with adjustment for age and sex. In addition, we assessed whether the PREDICT-PD risk score improved discrimination (C-statistics) and risk classification (net reclassification improvement) of incident PD beyond age and sex. During follow-up, 110 persons were diagnosed with incident PD. The PREDICT-PD risk score was associated with incident PD (hazard ratio [HR] = 1.30; 95 % confidence interval [1.06; 1.59]) and yielded a small, non-significant improvement in overall discrimination (ΔC-statistic = 0.018[−0.005; 0.041]) and risk classification (net reclassification improvement = 0.172[−0.017; 0.360]) of incident PD. In conclusion, the PREDICT-PD risk score only slightly improves long-term prediction of PD in the community.

**Electronic supplementary material:**

The online version of this article (doi:10.1007/s10654-016-0130-1) contains supplementary material, which is available to authorized users.

## Introduction

Parkinson Disease (PD) is the second most common neurodegenerative disorder among elderly [1]. At present, no treatment can effectively modify disease progression in patients with PD. This may be due to the advanced stage of pathology that PD patients already have at the time of clinical diagnosis [2]. The identification of persons from the general population who are at high risk of PD might open the door to earlier diagnosis, and possibly enable early symptomatic treatment. Equally important, it would enable the selection of persons who, possibly after additional refined screening, can be enrolled in neuroprotective trials.

In the most recent comprehensive meta-analysis of nonmotor features and risk factors for PD to date, several variables were determined to affect the risk of PD [3]. Subsequently, a cohort study in the United Kingdom was initiated (PREDICT-PD) to assess the validity of a risk score based on 11 of these variables to prospectively predict PD. In cross-sectional analyses at baseline, the PREDICT-PD risk score was associated with several proxies for PD [4]. However, the prospective usefulness of the PREDICT-PD risk score for PD remains unclear.

In this study, we investigated the prospective prognostic value of the PREDICT-PD risk score in an independent, population-based sample with 20 years of follow-up.

## Methods

### Study design and setting

The study was embedded in the first subcohort of the Rotterdam Study (RS-I), a large, prospective, population-based study in the Netherlands [5, 6]. The study was initiated in 1990, inviting all inhabitants of Ommoord who were aged ≥55 years. 7983 participants (78 %) agreed to participate and provided written informed consent. At baseline, participants were extensively screened for parkinsonism and dementia, and assessments of nonmotor features and risk factors used to derive the risk score were conducted [7, 8]. For this report, we excluded persons with prevalent parkinsonism or dementia and persons who were not screened for both, leaving 6492 persons for analyses. We followed participants for the development of PD from baseline until: onset of parkinsonism, onset of dementia, death or 1 January 2011, whichever came first. Until 2011, the study has had a total of five visits, including four follow-up visits. At each visit, participants underwent home interviews and medical examinations at the research center.

### Assessment of parkinsonism and PD

A detailed description of parkinsonism and PD assessment methods has previously been published [9] and is summarized in Online Resource 1. In short, we used four overlapping modalities to screen for potential parkinsonism during follow-up: in-person screening (on average every 4 years), in-person interviews, use of antiparkinson medication, and alerts from continuous monitoring of clinical records. For each modality, the proportion of incomplete data was small (range of averages: <1–12 %). Of all persons who screened positive in any of these methods, complete medical records (including letters from medical records of specialists and general practitioners) were studied and case reports were drawn up covering all potentially relevant information to establish presence and cause of parkinsonism. These case reports were evaluated by a panel led by an experienced neurologist. PD was only diagnosed after exclusion of secondary causes, and medical records of all incident parkinsonism cases (both PD and secondary) continued to be scrutinized until the end of the study period for new information that could lead to a revision of the diagnosis. Given the substantial overlap between the four detection methods we considered persons who were not screened in-person during one of the follow-up rounds still at risk for parkinsonism and PD. For onset of PD, we used the age at midpoint between the date on which parkinsonism first was observed (either during in-person screening or in medical records) and the preceding in-person examination. Person-time at risk for incident PD ended at onset of parkinsonism, incident dementia (date of clinical diagnosis), death, or January 1, 2011.

### Assessment of nonmotor features and risk factors in the PREDICT-PD risk score

Nonmotor features and risk factors used to derive the risk score were assessed during the baseline home interview and center visits. Smoking habits were assessed during home interviews and participants were subsequently categorized as current, former and never smokers. Coffee and alcohol intake were assessed using food-frequency questionnaires. In addition, participants were asked whether any of their parents, siblings or children had PD. Participants were also asked:”Did you ever have a serious head trauma or a concussion?” and “Did you ever have periods of depression?”.

During home interviews, participants were questioned for current medication they were using at the time. This included laxative medication, non-steroidal anti-inflammatory drugs (NSAIDs), calcium-channel blockers, beta-blockers, and other antihypertensive drugs (ATC-codes C02, C03, C07, C08, and C09). Since we had no data available on stool frequency, we considered use of laxative medication as a proxy for constipation. Blood pressure was measured twice during center visits, and hypertension was diagnosed if the mean of two measurements exceeded 140/90 mmHg or if a person used antihypertensive medication with an adequate indication.

We had no data on erectile dysfunction and consequently excluded erectile dysfunction from the risk score. In the meta-analysis, farming occupation, rural living, pesticide exposure, and well-water drinking were also identified as risk factors [3], but these factors were not included in the PREDICT-PD risk score [4]. In the Rotterdam Study, only 5 study participants (<0.1 %) worked as a farmer (none of whom developed PD during follow-up), and all study participants lived in a non-rural, suburban district (i.e., Ommoord). We lacked information on pesticide exposure and well-water drinking.

### Statistical analysis

We constructed a risk score for each individual, by adding up their number of risk factors weighted by the log-transformed, reported risk-increasing or (inverted) risk-decreasing effect size for the association with PD [3]. Risk scores were transformed into z-scores to facilitate evaluation of their effect per standard deviation increase. A higher risk score corresponds to a larger weighted number of risk factors and thus a higher expected risk of PD. We constructed two models: model I comprised age and sex for overall analyses, and only age for sex-stratified analyses. Model II comprised model I plus the PREDICT-PD risk score. We visually inspected reclassification of risk after addition of the PREDICT-PD risk score using a reclassification scatterplot [10].

We investigated the association between the risk score and incident PD by comparing model II to model I using the method proposed by Fine and Gray, which takes into account the risk of competitive events (i.e., incident dementia or death) [11]. We examined the interaction term of the PREDICT-PD risk score with sex, and subsequently stratified analyses by sex. The discriminative value of both models was expressed with Uno’s C-statistic, which takes into account right-censoring [12]. To study reclassification, we calculated the continuous net reclassification improvement (NRI) [13]. Since the predictive power of dependent-state risk factors may decrease over time, we repeated our prediction analyses after restriction of follow-up to the first 5 and 10 years, respectively.

We had complete data on 91 % of predictor values (missing values between 0 and 19 % per predictor). Missing values were handled by multiple imputation using the mean of five imputations, based on age, sex and all other nonmotor predictors.

## Results

The most prevalent nonmotor risk factors were coffee and alcohol use, while constipation and a family history of PD were the least prevalent (Table 1). During follow-up (87,321 person-years, median 16.1 years), 110 individuals had incident PD (age-adjusted incidence rate 1.4 per 1000 person-years) of whom 56 were men and 54 were women. In total, 3713 persons died, and 1021 were diagnosed with incident dementia while at risk of parkinsonism. In our population, the only risk factors that were independently associated with incident PD were current smoking, former smoking and depression (Table 1). As shown in Online Resource 2, women had effect estimates of laxative use, family history, hypertension, NSAID use, CCB use, and alcohol for incident PD that were direction-consistent with the meta-analysis, whereas men had opposite estimates. Furthermore, we observed a significant association between family history and incident PD in women, but not in men.

**Table 1:** Overview of population characteristics

Characteristic	N in the Rotterdam study	Reported RR/OR^a^	HR (95 % CI) in the Rotterdam study
Age at baseline, mean, y (SD)	68.7 (8.7)	–	1.03 (1.01; 1.05)
Women (%)	3818 (58.8)	–	0.39 (0.24; 0.62)
Smoking (%)			
Never	2202 (34.6)	1.00	
Former	2695 (42.4)	0.78	0.53 (0.32; 0.89)
Current	1463 (23.0)	0.44	0.36 (0.19; 0.67)
Family history (%)^b^	311 (5.0)	4.45	1.62 (0.80; 3.27)
Coffee (%)	5087 (97.2)	0.67	1.78 (0.38; 8.27)
Alcohol (%)	4154 (79.4)	0.90	0.87 (0.52; 1.44)
Hypertension (%)	3572 (55.0)	0.74	1.13 (0.74; 1.73)
NSAID use (%)	512 (7.9)	0.83	1.14 (0.58; 2.24)
CCB use (%)	388 (6.0)	0.90	1.42 (0.75; 2.69)
Beta-blocker use (%)	948 (14.6)	1.28	1.20 (0.72; 2.00)
Constipation (%)	237 (3.7)	2.34	1.35 (0.58; 3.13)
Head injury (%)	1980 (30.5)	1.58	0.77 (0.51; 1.18)
Self-reported periods of depression (%)	2028 (33.2)	1.86	1.63 (1.10; 2.42)

*N* number of persons at risk for Parkinson Disease, *RR* relative risk, *OR* odds ratio, *HR* hazard ratio adjusted for age, sex and all other risk factors, *95* *% CI*, 95 % confidence interval. *y* year, *SD* standard deviation, *NSAID* non-steroidal anti-inflammatory drug, *CCB* calcium channel blocker

For constipation, a proxy was used (use of laxative medication)

^a^Reported in the meta-analysis of early nonmotor features and risk factors by Noyce et al. [3]. Of note, no relative risks or odds ratios were reported for age and sex

^b^History of Parkinson Disease in parents, siblings or children

**Fig. 1:**
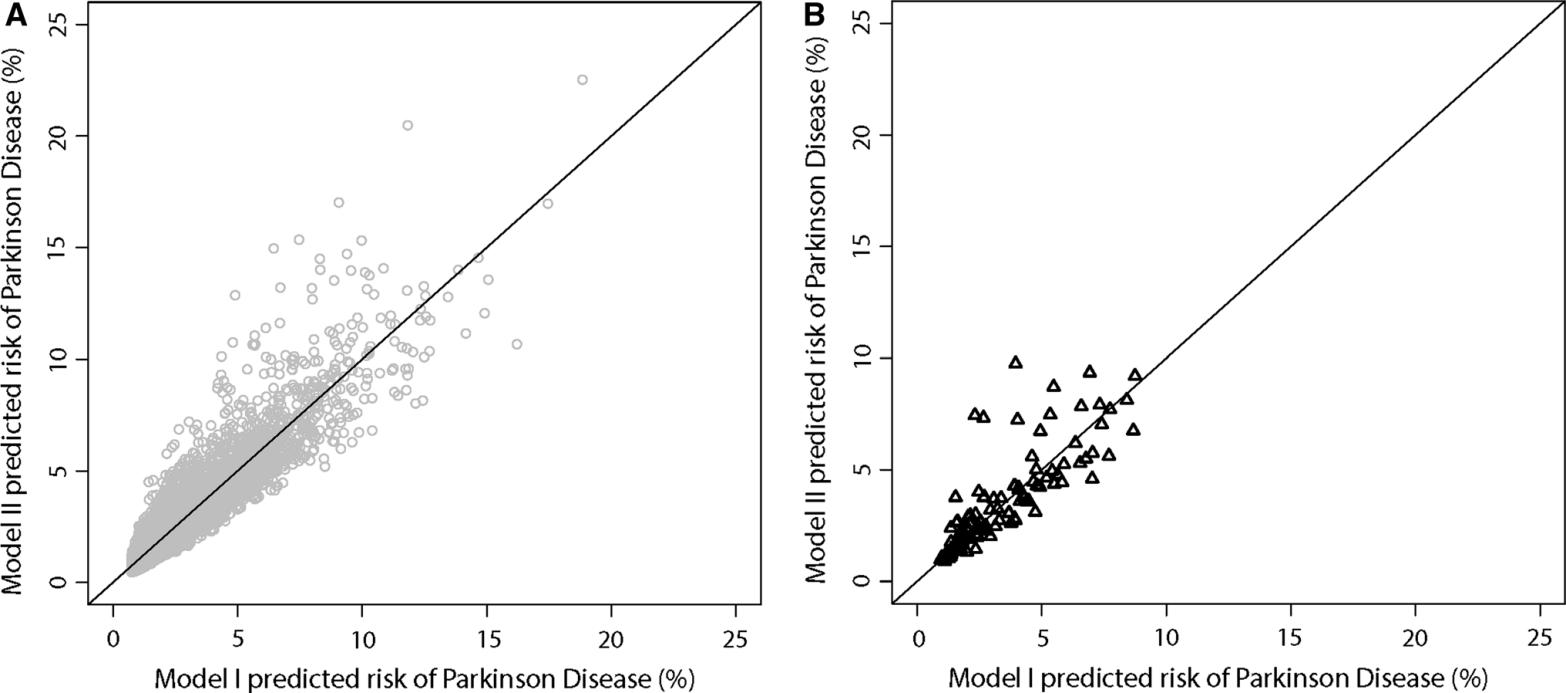
Reclassification scatterplot of the 20-year risk of incident Parkinson Disease after addition of the PREDICT-PD risk score. Model I, overall: age and sex. Model II, overall: age, sex and PREDICT-PD risk score. **a** Persons without incident Parkinson Disease. **b** Persons with incident parkinson disease

Predicted 20-year risk of PD ranged from 0.7 to 18.8 % in model I (median 2.2 %), and from 0.5 to 22.5 % (median 2.2 %) in model II (Fig. 1). During follow-up, persons in the highest PREDICT-PD risk score tertile consistently had the highest cumulative hazard of incident PD (Online Resource 3). The PREDICT-PD risk score was independently associated with incident PD and yielded a small, non-significant improvement in discrimination of incident PD beyond age and sex (Table 2; ΔC = 0.018 [−0.005; 0.041]). Compared to model I, model II slightly improved overall classification of PD risk.

**Table 2:** PREDICT-PD risk score and the 20-year risk of incident Parkinson Disease

Group	Model	Association	Discrimination	Reclassification
		*HR* *[95* *% CI]*	*C*-*statistic* *[95* *% CI]*	*NRI* *[95* *% CI]*
Overall	I	(Reference)	0.649 [0.592; 0.707]	(Reference)
	II	1.30 [1.06; 1.59]	0.667 [0.609; 0.725]	0.172 [−0.017; 0.360]
Men	I	(Reference)	0.684 [0.617; 0.752]	(Reference)
	II	0.90 [0.63; 1.30]	0.681 [0.605; 0.758]	−0.105 [−0.356; 0.145]
Women	I	(Reference)	0.604 [0.530; 0.677]	(Reference)
	II	1.70 [1.36; 2.12]	0.674 [0.602; 0.746]	0.461 [0.202; 0.721]

Model I, overall: age and sex. Model I, stratified analyses by sex: age

Model II, overall: age, sex and PREDICT-PD risk score. Model II, stratified analyses by sex: age and PREDICT-PD risk score

*HR* hazard ratio for incident Parkinson Disease per standard deviation in risk score. *CI* confidence interval. NRI, continuous net reclassification improvement (model I is reference)

The association between the PREDICT-PD risk score and incident PD was strongly modified by sex (p = 0.004). Stratified analyses showed that the risk score was associated with incident PD independently of age in women but not in men. In line with this, risk prediction of PD based solely on age was more accurate in men than in women, but this difference faded after application of the PREDICT-PD risk score. Classification of PD risk was improved by model II in women, but not in men.

After restriction of follow-up to 5 years, discrimination and risk classification of incident PD did not significantly improve (ΔC = 0.008 [−0.022; 0.037] and risk classification (NRI = 0.012 [−0.091; 0.145]) from model I to II. Similarly, after restriction of follow-up to 10 years, prediction did not improve (ΔC = 0.013 [−0.011; 0.038] and NRI = 0.031 [−0.069; 0.140]).

## Discussion

In this prospective, population-based sample with 20 years of follow-up, we found that the PREDICT-PD risk score yielded a small, non-significant improvement in overall discrimination and classification of incident PD. This was due to improvement of PD risk prediction in women to the level of men.

At present, there are no validated methods to identify persons at high risk for PD from the general population so that they can be monitored for onset of symptoms or enrolled in neuroprotective trials. The recently proposed PREDICT-PD risk score was based on a meta-analysis of early nonmotor features and risk factors [3, 4]. Strengths of our study were its prospective design and inclusion of community-dwelling individuals irrespective of PD risk. Compared to cross-sectional case–control data, such as from the multi-center Parkinson’s Progression Marker Initiative [14], prospective community-based studies such as the Rotterdam Study have the advantage that all participants (i.e., both PD future cases and controls) were included and followed up using the same methodology, presumably ensuring a realistic estimate of the risk of incident PD in the general population. Further strengths include long duration of follow-up for PD (median 16.1 years) and standardized assessment of PD diagnosis. In addition, our sample was completely independent of discovery samples used for relative risk estimates in the meta-analysis [3].

Limitations included lack of data on erectile dysfunction as well as the assessment of head trauma and depression using a single question. In addition, we used laxative medication as a proxy for constipation, which likely caused a severe underestimate of the true prevalence of constipation, since many people who suffer from constipation do not use drugs and change their dietary and lifestyle habits. In our sample, only a small proportion of male participants who did not develop incident PD and not a single male incident PD patient used laxatives at baseline, suggesting that our underestimate may have been larger in men than in women. If we would have had complete information on these factors, the PREDICT-PD risk score may have improved PD prediction significantly in our population. Furthermore, we lacked histologic confirmation of PD diagnosis, which may have introduced non-differential misclassification of PD cases. Also, we may have been underpowered to detect a small significant improvement in PD prediction, especially in the middle-long term (i.e., 5 years) The estimates used in the PREDICT-PD risk score were mostly based on studies that did not assess the majority of variables simultaneously, and the estimates were not sex-specific. In our sample, only 3 risk factors were independently associated with incident PD (current smoking, former smoking and depression), which may indicate that the meta-analyzed estimates were inflated due to limitations of the meta-analysis, such as publication bias, a substantial degree of selection in some discovery samples, or insufficient adjustment for covariates [3]. Alternatively, we may have been underpowered to detect significant associations with PD for separate risk factors, and limitations in our assessment methods may have led to underestimates of true associations. Future collaboration across cohort studies who have prospectively assessed (nearly) all risk variables in the score will probably increase the accuracy of risk estimates. Similarly, while we observed clear sex differences in associations between risk factors and incident PD, most of the sex-specific associations in our sample were non-significant. Collaborative studies may distinguish true sex differences from limitations in assessment methods that may have worse in men (e.g., laxative use).

Future studies can further build on the PREDICT-PD risk score by focusing on three other key aspects. First, some relatively common nonmotor risk factors for PD were not yet part of the risk score, such as impaired olfactory function [15]. Recently, dedicated olfactory function testing was shown to distinguish patients with a PD diagnosis from controls with very high accuracy [14]. Although the long-term prospective predictive value of olfactory testing for PD in the community has not yet been demonstrated empirically, a previous study showed that impaired olfaction is associated with PD up to 4 years before clinical diagnosis [15]. Therefore, inclusion of prospective measures of olfactory function in the risk score may further improve prediction of PD in the community. Second, while the Rotterdam Study comprises a suburban-based study population with only few farmers, discrimination and classification accuracy in other communities may be improved by inclusion of data on rural living and farming occupation. Third, motor features were not included in the risk score. Even in the absence of objective signs on routine screening, prediagnostic PD patients have subjective parkinsonian complaints more frequently than controls [16], and tremor is the most common presentation of PD patients in primary care practice 10 years before clinical diagnosis [17]. The advancement of dedicated motor screening tests might not only lead to reliably detection of PD in select subgroups of very high-risk persons (e.g., RBD-patients [18]), but potentially also in community-dwelling persons.

In conclusion, the PREDICT-PD risk score is a small step forward towards predicting incident PD in the community, in particular in women, but there is still a clear need for improvement.

## Electronic supplementary material

Below is the link to the electronic supplementary material.

Supplementary material 1 (DOCX 48 kb)
