# Supplementary material for: Predicting Parkinson disease in the community using a nonmotor risk score
Source: Eur J Epidemiol. 2016 Feb 22;31:679–84. doi: 10.1007/s10654-016-0130-1 (PMC4977330; doi:10.1007/s10654-016-0130-1)
Supplement: Supplementary file 1 — Supplementary material 1 (DOCX 48 kb) [file 10654_2016_130_MOESM1_ESM.docx]

**Online Resources:**

**Predicting Parkinson Disease in the community using a nonmotor risk score**

Sirwan K.L. Darweesh, MD, MSc^1^ [s.darweesh@erasmusmc.nl](mailto:s.darweesh@erasmusmc.nl)

Peter J. Koudstaal, MD, PhD^,2^ [p.j.koudstaal@erasmusmc.nl](mailto:p.j.koudstaal@erasmusmc.nl)

Bruno H. Stricker, MB, PhD^1,3^ [b.stricker@erasmusmc.nl](mailto:b.stricker@erasmusmc.nl)

Albert Hofman, MD, PhD ^1^ [a.hofman@erasmusmc.nl](mailto:a.hofman@erasmusmc.nl)

Ewout W. Steyerberg, PhD^4^ [e.steyerberg@erasmusmc.nl](mailto:e.steyerberg@erasmusmc.nl)

M. Arfan Ikram, MD, PhD ^1,2,5*^ [m.a.ikram@erasmusmc.nl](mailto:m.a.ikram@erasmusmc.nl)

^1^ Department of Epidemiology, Erasmus MC University Medical Center Rotterdam, Rotterdam, the Netherlands

^2^ Department of Neurology, Erasmus MC University Medical Center Rotterdam, Rotterdam, the Netherlands

^3^ Inspectorate for Health Care, The Hague, the Netherlands

^4^ Department of Public Health, Erasmus MC University Medical Center Rotterdam, Rotterdam, the Netherlands

^5^ Department of Radiology, Erasmus MC University Medical Center Rotterdam, Rotterdam, the Netherlands

*Corresponding Author:

M. Arfan Ikram, MD, PhD, Department of Epidemiology, Erasmus MC University Medical Center, Dr. Molewaterplein 50, 3015 GE, Rotterdam, the Netherlands; Telephone +31 10 704 34 88; Fax +31 10 704 46 57; Email [m.a.ikram@erasmusmc.nl](mailto:m.a.ikram@erasmusmc.nl)

Requests for reprints should be addressed to M. Arfan Ikram, MD, PhD.

**Online Resource 1. Ascertainment methods of parkinsonism and Parkinson Disease**

*Overview of detection modalities*

We used four overlapping modalities to detect potential parkinsonism patients during follow-up: in-person screening, self-reported Parkinson Disease (PD) during in-person interviews, antiparkinsonian medication use, and clinical monitoring alerts. Of all persons who were detected by any of these methods, complete medical records were studied and case reports were drawn up covering all potentially relevant information (i.e., both information from in-person examinations and medical records) to establish the degree of certainty and subtype of parkinsonism. These case reports were evaluated by a panel led by an experienced neurologist using consistent criteria.

Participants were screened every four years using the same two-phase design.[9] First, participants were extensively screened by trained research assistants using standardized, bilateral assessments, including tremor (resting, positional and intentional), hypo- and bradykinesia (arm swing, gait, finger tapping, general impression), cogwheel rigidity, and postural reflex. Persons who screened positive were invited for a structured physical examination by a research physician specialized in neurologic diseases. Those who screened positive received a structured workup by a research physician specialized in neurologic disorders to establish parkinsonism. The average participation for in-person screening was 88% per round. Furthermore, in-person interviews were conducted at multiple center visits by trained research assistants asking the following questions: “Do you have Parkinson Disease?”; “Do you use drugs for Parkinson Disease?”; ”What was the age of diagnosis?”; “Have you been treated for Parkinson Disease by your general practitioner and/or specialist?” We considered all persons who said to have PD screen-positive, and we studied their complete medical records. The average participation for in-person interviews was 95% per round.

In addition, the cohort was continuously monitored for detection of new PD cases through a surveillance system by computer linkage with the general practitioners’ automated medical record systems, which encompasses free text entries by general practitioners as well as documentation from neurologists, geriatricians, and other medical specialists. Moreover, nearly all participants in this study were registered at one or more of the seven community pharmacies that serve the study area, and there was an automated link of the study database with pharmacy records. This makes it possible to identify subjects who used antiparkinsonian medication at any time during follow-up. Information on clinical alerts, including in persons who were institutionalized, was nearly complete throughout study follow-up (>99% completeness of follow-up), as were pharmacy records on antiparkinsonian medication use (ATC-code N04).

Given the substantial overlap between the four detection methods and the consistently high response rates for follow-up in-person examinations, we considered persons who were not screened in-person during one of the follow-up rounds still at risk for parkinsonism and PD.

Of all persons who were detected by any of these methods, complete medical records were studied and case reports were drawn up covering all potentially relevant information to establish the degree of certainty and subtype of parkinsonism. These case reports were evaluated by a panel led by an experienced neurologist using the criteria listed below. After initial diagnosis, medical records of all incident parkinsonism cases (both Parkinson Disease and secondary) continued to be scrutinized until the end of the study period for new information that could lead to a revision of the diagnosis.

*Diagnostic criteria for parkinsonism*

- Definite: detailed account of observation of bradykinesia plus at least one other cardinal sign (resting tremor, rigidity, postural instability) by any physician.
- Probable: multiple observations of parkinsonism by any physician without explicit description of bradykinesia and/or other cardinal signs, or a single observation of parkinsonism without explicit description of bradykinesia and/or other cardinal signs by a physician with extensive experience with parkinsonism (such as a neurologist or experienced geriatrician).
- Possible: single account of observed parkinsonism by any physician

*Diagnostic criteria for Parkinson Disease*

Participants with probable or definite parkinsonism, in whom all causes of secondary parkinsonism as well as dementia before the onset of parkinsonism had been excluded, and at least one of the following:

- A good response to antiparkinsonian medication (i.e. one or more signs improved while treated with antiparkinsonian medication)
- DaTSCAN findings consistent with Parkinson Disease
- Diagnosis of Parkinson Disease confirmed by a neurologist

We note that we had no DaTSCAN-data for any of the incident PD cases in the current dataset (which covered follow-up until January 2011).

*Diagnostic criteria for secondary parkinsonism*
The diagnostic criteria for secondary parkinsonism (e.g. secondary to dementia, medication-induced, vascular, multiple system atrophy, progressive supranuclear palsy) have previously been reported in detail.[7, 8] In short, participants were screened for dementia at baseline and during follow-up examinations using a three-step protocol,[8] comprising two brief tests of cognition to screen all subjects and the Cambridge Examination for Mental Disorders of the Elderly in individuals with positive screen results. Additional information was obtained from in-person examination by a neuropsychologist, continuous clinical monitoring of the entire cohort and neuro-imaging. A consensus panel, led by a neurologist, decided on the final diagnosis in accordance with standard criteria using the DSM-III-R criteria for dementia. Furthermore, the cohort was continuously monitored for detection of new stroke cases. For parkinsonism related to cerebrovascular disease, there had to be a clear time relationship between a cerebrovascular event and onset of atypical parkinsonism, usually without tremor, preferably supported by neuroimaging. For medication-induced parkinsonism, a person had to have used neuroleptic or other anti-dopaminergic drugs in the six months preceding the onset of parkinsonism and had to be without history of parkinsonism. Diagnosis of parkinsonism due to other secondary causes relied on specific information on essential features of each cause (e.g. vertical gaze palsy for progressive supranuclear palsy; concomitant autonomic and cerebellar dysfunction for multiple system atrophy).

Finally, persons who did not meet diagnostic criteria for any of the above causes of parkinsonism, persons with more than one potential cause, and persons for whom secondary causes of parkinsonism could be excluded but who did not respond to antiparkinsonian drugs were classified as having unspecified parkinsonism. After initial diagnosis, medical records of all incident parkinsonism cases (both PD and secondary) continued to be scrutinized until the end of the study period for new information that could lead to a revision of the diagnosis.

**Online Resource 2. Overview of population characteristics by Parkinson Disease status and sex**

|  | **Males** | | | **Females** | | |
| --- | --- | --- | --- | --- | --- | --- |
|  | **Incident PD**  **(n=56)** | **No incident PD**  **(n=2618)** | **HR (95% CI) in the Rotterdam Study** | **Incident PD**  **(n=54)** | **No incident PD**  **(n=3764)** | **HR (95% CI) in the Rotterdam Study** |
| Age at baseline, mean, y (SD) | 72.1 (12.5) | 66.8 (11.4) | 1.05 (1.02; 1.08) | 70.5 (13.1) | 68.4 (13.8) | 1.01 (0.98; 1.04) |
| Smoking (%) |  |  |  |  |  |  |
| Never | 8 (15.1) | 210  (8.1) | 1.00 | 40 (74.1) | 1944  (53.0) | 1.00 |
| Former | 32 (60.4) | 1608 (62.3) | 0.48 (0.21; 1.10) | 12 (22.2) | 1043 (28.4) | 0.58 (0.30; 1.10) |
| Current | 13 (24.5) | 765 (29.6) | 0.43 (0.17; 1.10) | 2 (3.7) | 683 (18.6) | 0.15 (0.03; 0.64) |
| Family history (%)** | 2 (3.8) | 104  (4.1) | 1.08 (0.31; 3.73) | 6 (11.5) | 199  (5.5) | 2.26 (0.96; 5.31) |
| Coffee (%) | 42 (97.7) | 2064 (97.7) | 2.05 (0.11; 36.97) | 42 (97.7) | 2939 (96.8) | 1.76 (0.31; 9.89) |
| Alcohol (%) | 37 (86.0) | 1851 (87.6) | 1.12 (0.44; 2.88) | 28 (65.1) | 2238 (73.7) | 0.68 (0.37; 1.26) |
| Hypertension (%) | 37 (67.3) | 1364  (53.0) | 1.52 (0.84; 2.73) | 32 (59.3) | 2139 (57.6) | 0.87 (0.47; 1.61) |
| NSAID use (%) | 5 (8.9) | 139  (5.3) | 1.71 (0.66; 4.41) | 5 (9.3) | 363  (9.6) | 0.84 (0.33; 2.15) |
| CCB use (%) | 9 (16.1) | 172  (6.6) | 2.24 (1.03; 4.86) | 2 (3.7) | 205  (5.4) | 0.52 (0.13; 2.12) |
| Beta-blocker use (%) | 12 (21.4) | 386  (14.8) | 1.16 (0.57; 2.36) | 9 (16.7) | 541 (14.4) | 1.20 (0.56; 2.56) |
| Constipation (%) | 0 (0.0) | 48  (1.8) | 0.00* | 6 (11.1) | 183  (4.9) | 2.08 (0.87; 4.99) |
| Head injury (%) | 11 (20.8) | 913  (35.3) | 0.54 (0.29; 1.01) | 17 (31.5) | 1039 (28.0) | 1.08 (0.61; 1.93) |
| Self-reported periods of depression (%) | 14 (28) | 656  (26.2) | 1.16 (0.64; 2.09) | 29 (56.9) | 1329 (37.9) | 2.23 (1.24; 4.01) |

HR, hazard ratio adjusted for age, sex and all other risk factors. 95% CI, 95% confidence interval.

N, number of persons at risk for Parkinson Disease. RR, relative risk. OR, odds ratio. y, year; SD, standard deviation. NSAID, non-steroidal anti-inflammatory drug. CCB, calcium channel blocker.

For constipation, a proxy was used (use of laxative medication).

*Since there were 0 cases in the male incident PD stratum, no stable confidence interval was obtained.

**Online Resource 3. Survival without Parkinson Disease by PREDICT-PD risk score tertile**


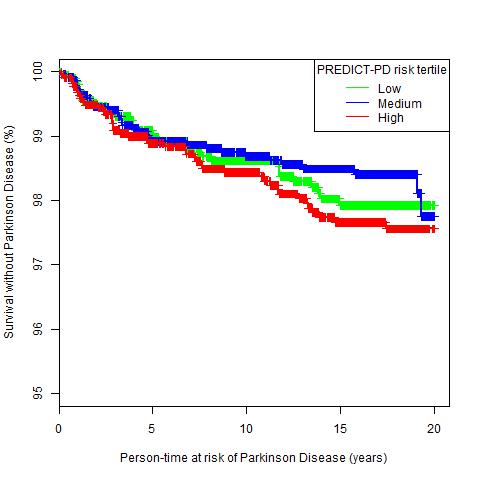


Y-axis denotes the percentage of persons surviving who remain free of Parkinson Disease
